# A Comparative Ex Vivo Study on Apex Locator Precision in Mature Teeth and Immature Teeth With Divergent Canals

**DOI:** 10.7759/cureus.69116

**Published:** 2024-09-10

**Authors:** Noam Kaufman, Avia Fux Noy, Iris Slutzky-Goldberg

**Affiliations:** 1 Prosthodontics, Hebrew University of Jerusalem, Jerusalem, ISR; 2 Pedodontics, Hebrew University of Jerusalem, Jerusalem, ISR; 3 Endodontics, Galilee College of Dental Sciences, Nahariya, ISR

**Keywords:** tooth development, immature teeth, ex vivo, electronic apex locators, accuracy

## Abstract

Aim: This study investigates the accuracy of an apex locator in mature and immature teeth with divergent root canals ex vivo.

Materials and methods: Fifty extracted premolar teeth were utilized for the study, with 25 mature teeth (Nolla stage 9 or 10) and 25 immature teeth (Nolla stages ≤ 8). The discrepancies between the actual length (AL) and the electronic length (EL), measured using a Bingo 1020 apex locator, were compared and analyzed. Statistical analysis included Pearson's correlation analysis, a T-test for independent samples, and multiple linear regression. Statistical significance was set at p < 0.05.

Results: The difference between AL and EL in immature teeth was significantly higher than in mature premolar teeth. Nonetheless, patient gender or jaw type (upper/lower) did not affect the accuracy of electronic apex locator (EAL) measurements.

Conclusion: EAL readings are less accurate in immature teeth than in mature teeth. Supplementary measures, such as radiographic length determination and wet-dry paper point tests, are required to confirm the working length for improved treatment outcomes and patient compliance.

## Introduction

Root canal therapy aims to treat and prevent apical periodontitis [1]. Cleaning and shaping the root canal is essential for the success of endodontic treatment. The importance of working within the confines of the root canal without overextension or under extension of the root filing has already been demonstrated [1,2]. The use of electronic apex locators (EALs) allows for accurate determination of the root canal length [3]. However, certain factors may affect the accuracy of a test result, such as the presence of inflammatory exudate or blood [3]. Using a Sono-Explorer (Hayashi Dental Supplies, Japan), a second-generation apex locator, it was found that a wide apical foramen negatively affected the apex locator accuracy [3]. Unreliable apex locator readings were observed when the size of the apical constriction was larger than an ISO size 100 [4]. In an in-vitro study, the accuracy of the Root ZX (Morita, Japan) was calculated for apical foramina enlarged to sizes 60-100. The EAL accuracy in teeth with an apical size of >80 dropped significantly, even when the tolerance level was 1 mm [5]. Similar results were obtained in another study, demonstrating that the measurement error increased in teeth with larger apical diameters (size: 150). The authors indicated that shorter readings were obtained when small-sized files were used for length determination and that the type of apex locator used significantly affected the result [6].

Length determination is essential in root canal treatment of immature teeth during apexification or regenerative procedures [7]. It is necessary to exercise caution during a child's treatment as pain caused by the passage of a file beyond the apex can increase anxiety, reduce trust, and decrease the cooperation of the patient. Taking all necessary measures to prevent such incidents is crucial [8]. In immature teeth, the canals are wide and blunderbuss, making it difficult to determine the working length, either radiographically or with the aid of an EAL, because an apical constriction has not yet formed [9]. The dentinal tubules in immature teeth are wider and become narrower with age [10], and this may have an influence on EAL readings. Previous studies used mature teeth in which the apical foramina was mechanically enlarged to simulate immature teeth [11], but the root canals in immature teeth are not only wider but also divergent [9]. Therefore, these studies can be effective in demonstrating EAL readings in teeth with apical root resorption or when the development of the root is almost complete (Nolla stage 9), but not in less developed apices. Furthermore, the precise location of the cemento-dentinal junction in immature teeth is poorly defined.

The aim of this study is to determine, by comparing readings in mature and immature teeth, whether EAL is affected by root development. The null hypothesis is that EAL readings in immature teeth are as accurate as readings in mature teeth.

## Materials and methods

Premolar teeth extracted in the oral surgery department for periodontal or orthodontic reasons were used in this ex vivo study. The study was conducted at Hadassah Medical Center, Jerusalem, Israel. By signing a written consent form, the patients or their parents approved the use of their teeth for this study. Only teeth with one or two straight roots were included in the study, while teeth with irregular anatomy or three-root canals were excluded from the study. To determine their suitability for the study, radiographs of all the teeth were taken from the mesiodistal and buccolingual directions.

The study included 50 premolar teeth with 67 canals, taken from 36 patients. Twenty-two men (58%) and 14 women (42%) participated in the study. The patients' age range was 10-67 years, and the mean age was 31.82 years (SD = 22.14). All the extracted teeth were kept in a glass container with physiological water at a temperature of 4℃. The teeth were divided into two groups according to their stage of root development, based on Nolla classification [9], with group 1 consisting of 25 teeth at a development stage of 9 or 10, and group 2 consisting of 25 teeth at a development stage ≤ 8. The sample size was calculated assuming a 95% confidence level and a 5% margin of error.

The technique described by Shacham et al. [11] was used to measure the working length of the tooth. First, the length of the tooth was measured externally in millimeters (tooth length (TL)). Then, the canal orifice was gradually enlarged using Gates Glidden drills (sizes: 2 to 4) (Mani® Inc., Utsunomiya, Japan). K-files (Mani® Inc., Utsunomiya, Japan) were inserted in ascending order until binding was achieved, and the tip of the file was visible at the apical foramen. This length was recorded as the actual length (AL). The teeth were then embedded in freshly mixed alginate (Aroma fine plus fast set, GC Co., Tokyo, Japan). The canals were filled with 3% sodium hypochlorite, and excess irrigant was removed from the pulp chamber using a cotton pellet. The length was determined using a Bingo 1020 apex locator (Forum Technologies, Rishon Lezion, Israel), which was connected to the file positioned earlier in the AL test and to a hook embedded in the alginate. Each tooth was measured three times, and the mean electronic length (EL) was calculated. The lengths obtained in the readings (EL, AL, and TL) were compared for each tooth. Then, a comparison was made between the AL-EL difference and the stage of root development, the patient's age, gender, and the jaw.

The results were compiled into an Excel table. The data were statistically processed using IBM SPSS Statistics for Windows, Version 28 (Released 2021; IBM Corp., Armonk, New York, USA). Statistical analysis included Pearson's correlation analysis, a T-test for independent samples, and multiple linear regression. Statistical significance was set at p < 0.05. This study was approved by the Helsinki Institutional Committee (HMO-21-0583).

## Results

The teeth included in the study were distributed as follows: group 1 included mature teeth from patients aged 16-67 years (mean age: 50.92, SD = 15.46); Group 2 included immature teeth from patients aged 10-15 years (mean age: 12.72, SD = 1.13). Twenty-six (52%) of the premolars were from the maxilla, and 24 (48%) from the mandible; 28 (56%) of the teeth were first premolars, and 22 (44%) were second premolars; 25 teeth were included in each group. Group 1 included six teeth with two canals, whereas group 2 included 11 teeth with two canals. Table 1 displays the distribution of the teeth.

**Table 1:** The distribution of the teeth in the study

Variables	Values	Frequency	Percent
Gender	Male	22	61%
	Female	14	39%
Jaw	Upper	26	52%
	Lower	24	48%
Premolar	First	28	56%
	Second	22	44%
Apex	Immature	25	50%
	Mature	25	50%

Quantitative clinical variables

The measurements of the TL, AL, and EL, and the difference between AL and EL (DIF) are presented in Table 2.

**Table 2:** Central indices, dispersion of the study variables N = 67; lengths in mm

Variables	Max	Min	Mean	SD
Tooth length (TL)	27	16.5	20.19	2.64
Actual length (AL)	26.5	16.5	20.51	2.66
Electronic length (EL)	26	15	19.88	2.79
DIF (AL-EL)	1.5	-0.5	0.63	0.43

Multiple linear regression was conducted using the Enter method to predict the accuracy of the EAL using clinical and demographic variables. The model was found to be statistically significant (F{5,44} = 3.827, p = 0.006, R² = 30.3%). A statistically significant relationship was found between the accuracy level of the EAL and age (p = 0.006, β = 0.753): the younger the patient was, the higher the DIF.

Statistical inference regression was conducted to predict the DIF using the clinical and demographic variables. Table 3 displays the correlation test results among the research variables.

**Table 3:** Prediction of the DIF using the clinical and demographic variables ^*^: statistically significant - p < 0.05; DIF: difference between actual length and electronic length

Variables	B	β	p
(Constant)	1.094		0.001^*^
Age in years	0.014	0.753	0.006^*^
Gender	0.101	0.126	0.345
Upper/lower jaw	-0.011	-0.015	0.911
First/second premolar	0.157	0.197	0.154
Apex: mature/immature	-0.805	-1.019	0.001^*^

Pearson correlations between the dependent research variables revealed the following significant positive relationships: between TL and AL (r = 0.992, p < 0.001), between TL and EL (r = 0.981, p < 0.001), and between EL and AL (r = 0.989, p < 0.001) (^**^p < 0.01).

Pearson correlations between age and DIF were assessed. No significant relationship was found between the variables (r = -0.100, p = 0.224). T-tests were conducted for independent samples with the following variables: gender, jaw, first or second premolar, and the degree of root development. No significant difference was found between male and female patients (mean: 0.60 ± 0.34, 0.76 ± 0.46, respectively) with reference to DIF (T{48} = -1.399, p = 0.084). No significant difference was found between the maxillary and mandibular teeth (mean: 0.67 ± 0.45, 0.66 ± 0.35, respectively) and DIF (T{48} = 0.056, p = 0.478). No significant difference was found between the results obtained in the first premolars (0.62 ± 0.40) and second premolars (0.73 ± 0.40) and DIF (T{48} = -0.898, p = 0.187). A statistically significant relationship was found between the accuracy level of the EAL and the degree of root development (p < 0.001, β = -1.019): the larger the diameter of the apex was, the higher the DIF.

Since immature and mature teeth had different root lengths, to ensure a valid comparison of the results, it was necessary to focus on the difference between measurements for each tooth. Therefore, we compared the difference (∆ AL-EL) rather than AL and EL. The measurements obtained for the teeth in both groups are presented in Table 4.

**Table 4:** A comparison between the measurements in immature and mature teeth The results of the measurements made in both groups are presented in millimeters. T-tests were conducted for independent variables, p < 0.05. ^*^: significance found was p = 0.01 AL: actual length; EL: electronic length

	AL		EL		∆ AL-EL	
	Mean	SD	Mean	SD	Mean	SD
Immature teeth	18.417	0.874	17.653	1.013	0.764^*^	0.348
Mature teeth	22.952	1.791	22.468	1.756	0.484^*^	0.456

**Figure 1:**
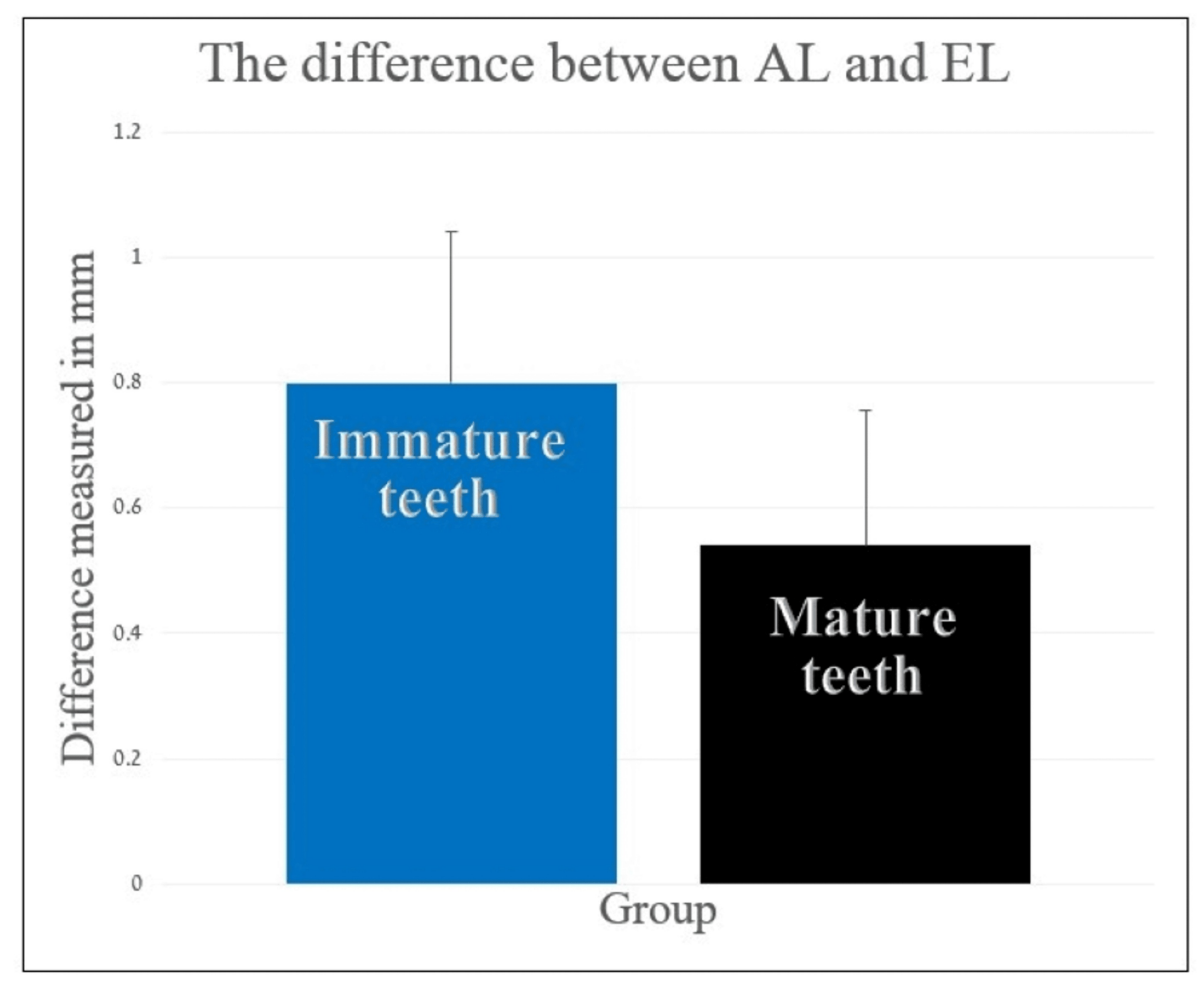
Comparison of the difference between actual length and electronic length of mature and immature (Nolla ≤ 8) teeth The difference between the groups was statistically significant (p ≤ 0.05). AL: actual length; EL: electronic length

The DIF in immature premolars (mean: 0.764± 0.384) was higher than the DIF in mature or almost mature premolars (mean: 0.484 ± 0.456) (T{48} = 2.414, p = 0.010) (Figure 1).

**Figure 2:**
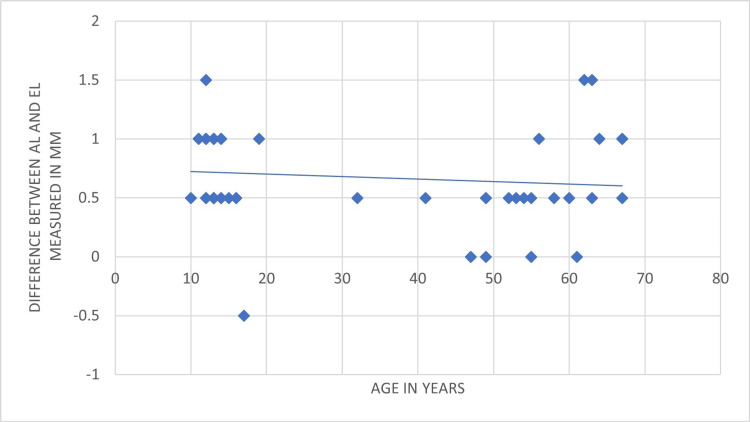
Patients' ages and the accuracy of apex locators reading More accurate readings were observed in older patients. The lengths were measured in millimeters. AL: actual length; EL: electronic length

The correlation between the accuracy of the EAL and patients' age is presented in Figure 2.

In terms of the association between the patient's age and the accuracy of the EAL, the accuracy of the apex locator increased when used on older patients. Less accurate readings were observed in younger patients.

## Discussion

Clinicians have used EAL for many years during root canal treatment to determine the working length. It is widely accepted that EAL provides better accuracy than radiographic assessment [3]. Root ZX has been considered the gold standard of apex locators for many years due to its 90% accuracy within a tolerance of 0.5 mm [3]. The Bingo 1020, used in this study, was as reliable and accurate as the Root ZX [12,13]. However, there are doubts about the accuracy of EAL readings in immature teeth [4-6,11].

The difference between AL and TL was statistically different between the two study groups (Figure 1). This implies that the readings of the EAL in immature teeth are less accurate than those in mature teeth. However, the degree of accuracy was not influenced by the patient's gender, jaw type, or type of premolar. These findings are consistent with previous papers showing inaccurate readings on teeth with wide foramina [5,6,11]. The observed difference was statistically significant (p = 0.01), which means that the null hypothesis can be rejected (Figure 1). These results are in accordance with a previous work that compared the EAL reading in teeth at the beginning of an apexification procedure to those in teeth that completed apexification and found inaccurate readings only in immature teeth [14].

Inaccurate readings of EAL in immature teeth call for additional measures for length determination, such as using the tactile method or paper-point technique and relying more on the radiographic length determination [15,16]. An in vivo study that compared the tactile method, apex locator readings, and conventional radiographs in permanent multirooted teeth did not find any statistical difference between the methods. However, it included multirooted teeth with mature apices [15]. A study that tested the effect of the canal diameter on the EAL accuracy found that the accuracy of the apex locator was 95% when files sized 70 or more were used in teeth with an apical diameter of 0.8 mm, but not in larger sizes [5]. Another study that examined the accuracy of the Apit 11 apex locator (Osada Electric Co., Tokyo, Japan) in canals enlarged to sizes 60-80 found accurate readings in such canals, although the accurate readings were obtained only when the size of the file was smaller than canal diameter by no more than 0.2 mm [11]. Unlike different definitions of the immature roots by the size of the apical foramen, which according to ISO sizes is larger than 40-100 [4], the definition of the immature apex according to Nolla’s, Cvek’s or Moorrees’ classification refers to the shape of the apical canal, be it open or closed, convergent, parallel or divergent [9,17-19].

**Figure 3:**
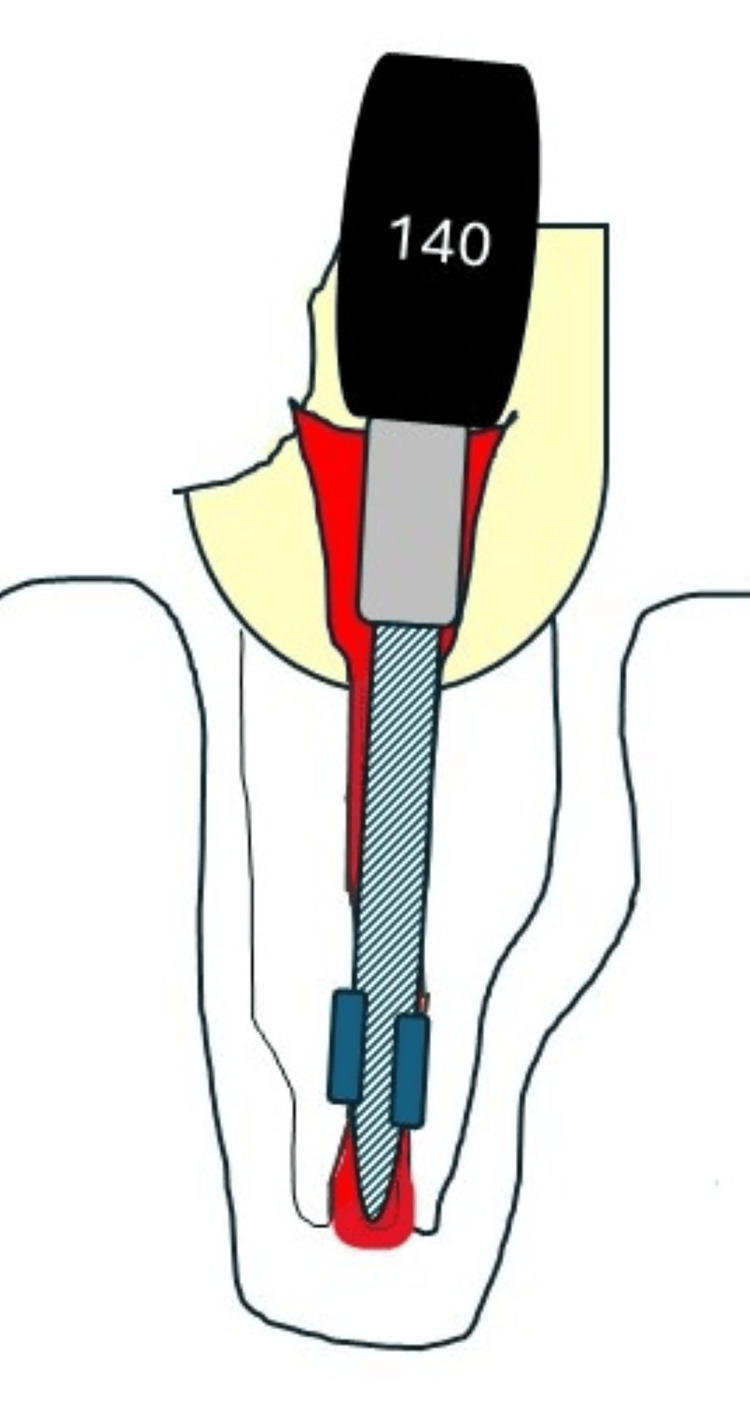
Binding of a file in a divergent root canal Binding of the file in a divergent root canal is expected to occur 2-3 mm coronal than the apical foramen. Credit: Iris Slutzky-Godlberg

The binding of the file occurs in the narrowest area in the canal, that is, 2-3 mm coronal to the apical foramen because the size of the apical foramen is larger than the diameter at the binding point, and the difference would be larger than 0.2 mm. This may have an impact on EAL readings (Figure 3).

Studies that use serial enlargement of the apex [5,11] only replicate convergent roots and do not accurately mimic the immature apex. In younger patients, the roots are less developed and the apical canal is more divergent. The results of the current study demonstrated lower accuracy of the EAL in younger ages (Figure 3). This may also affect the accuracy of apex locators in immature roots. The tolerance level for the EAL readings was set at 0.5 mm. While larger tolerance levels (i.e., ±1 mm) may improve the accuracy of the EAL test [11], they can also cause over- or under-instrumentation, or even an apical perforation through the apex [11]. A study that compared the accuracy of three apex locators, Root ZX, iPex, and YS-RZ-A, in teeth with immature apices demonstrated high accuracy levels when the tolerance was <1 mm, but the results were much less accurate when the direct and electronic measurements were compared (53.3%, 33.3%, and 26.3%, accordingly) [19,20].

Determining the length of the root canal is essential to ensure that the instrumentation and obturation are confined to the canal's limits [3]. It has been well established that length determination during root canal treatment and its maintenance are among the factors influencing the outcome of the treatment [1]. Inadvertent extrusion of irrigants and obturation material into the periapical tissues may result in pain, inflammation, and periapical disease [2,19,20]. In immature teeth, this factor may have an additional effect when placing an apical plug before root filling. In a study of non-vital traumatized immature teeth, mineral trioxide aggregate (MTA) plugs were more often inaccurately placed in divergent roots than in convergent roots. The position of the apical barrier was found to influence the outcome after two years, depending on the location of the plug [21].

Post-operative pain following endodontic treatment is common and can be caused by a range of factors, including chemical, mechanical, microbial, and host-related damage that occurs during the treatment. This pain has a potential pathogenic link to the acute inflammation of the periapical area caused by localized damage [22]. Patients undergoing endodontic therapy may experience increased intra-operative pain, which is associated with various factors, such as pulp status, gender, age, tooth type, and length of treatment [23-25].

Pain resulting from over-instrumentation and extruded filling materials may affect the future cooperation of young patients. An unpleasant experience with poorly managed pain during dental treatment can cause patients to avoid seeking further treatment and make it more difficult to treat. Pain management is particularly important in pediatric dentistry, as patient perceptions of dental treatment are established during childhood. Pain and negative experiences during dental treatment are major reasons for dental fear, anxiety, and behavior management problems in young patients. It is important for dental professionals to acknowledge this fact to prevent pain and discomfort [26].

Moreover, traumatic dental injuries (TDIs) are a significant concern in pediatric dentistry due to the high frequency of TDIs among children. It is particularly noteworthy that there is a peak in TDI frequency at the age of eight [27], as at this age, children's permanent incisors are still developing with incomplete root formation and apical diameters that may be even wider than the largest endodontic file available (ISO size ≥ 140). Severe dental trauma often leads to pulp necrosis, especially in cases of luxation injuries. When pulp necrosis occurs, it requires endodontic intervention. Given the high frequency of TDIs, it is crucial to accurately measure the TL and apply the appropriate method accordingly.

The primary limitation of this study lies in its relatively small sample size. Future research should aim to validate the findings of this study by utilizing different types of apex locators in a larger and more diverse population cohort in an in vivo setting.

## Conclusions

The accuracy of the Bingo 1020 EAL readings was lower in immature teeth compared to mature teeth, possibly due to the divergent shape of the canal causing premature file binding. Factors such as gender, jaw, and type of tooth did not affect EAL readings. However, patients' age significantly affected the accuracy of the reading due to the developmental stage of the tooth. It is recommended to use supplementary measures, such as paper points, to confirm the actual working length for improved treatment outcomes and patient compliance.
